# Rare Bilateral Massive Pneumothorax, Pneumomediastinum, Pneumoperitoneum, and Diffuse Subcutaneous Emphysema During Colonoscopy: Multidisciplinary Collaboration: Case Report

**DOI:** 10.1155/crgm/6444675

**Published:** 2025-09-19

**Authors:** Li-Li Liu, Mai-Qiao Yang, Rui Wu, Bing-Xing Li

**Affiliations:** ^1^Department of Anesthesiology, Yan'an Hospital Affiliated to Kunming Medical University, Kunming, Yunnan, China; ^2^Department of Digestive Endoscopy, Yan'an Hospital Affiliated to Kunming Medical University, Kunming, Yunnan, China

**Keywords:** ESD colonic, pneumomediastinum, pneumoperitoneum, pneumothorax, SCE

## Abstract

While colonoscopy is generally considered to be a safe procedure, serious complications such as intestinal perforation may also occur. Herein, we describe an extremely rare clinical case of acute colonic perforation during colonoscopy treatment. A 55-year-old female patient with good health presented to our hospital with abdominal pain for 2 months. While undergoing endoscopic submucosal dissection (ESD) of a colonic polyp, the patient developed sudden abdominal distension and dyspnea. An emergency computed tomography (CT) scan was performed and demonstrated bilateral massive pneumothorax, pneumomediastinum, pneumoperitoneum, and generalized subcutaneous emphysema (SCE). The patient underwent gas extraction, bilateral intercostal pneumothorax drainage, and conservative medical management. The patient had a favorable postoperative course and was discharged home on day 14. This case report highlights the clinical rarity of gas extravasation complications during colonoscopy and underscores the importance of multidisciplinary collaboration for accurate diagnosis and effective management, thereby avoiding surgical procedures.

## 1. Introduction

ESD, recognized worldwide as a minimally invasive treatment for mucosal lesions, provides favorable and precise solutions for gastrointestinal mucosal lesions but with an incidence of complications such as perforation [1]. Although perforation-induced gas extravasation can migrate to multiple embryo-related body compartments, it is extremely rare for bilateral pneumothorax, mediastinal emphysema, pneumoperitoneum, and generalized subcutaneous emphysema (SCE) to occur at the same time. Monitoring of the patient during the procedure is initially clinical, and the need for further imaging postprocedure should be based on clinical assessment. CT is the best imaging modality for confirming the diagnosis of pneumoperitoneum or pneumoretroperitoneum and may often localize the source. CT with rectal contrast may also be helpful for localizing the site of perforation in the large bowel. We report an unusual case of colonic perforation with the aim of highlighting the importance of multidisciplinary teamwork in the management and treatment of this condition and associated complications.

## 2. Case Report

**Figure 1:**
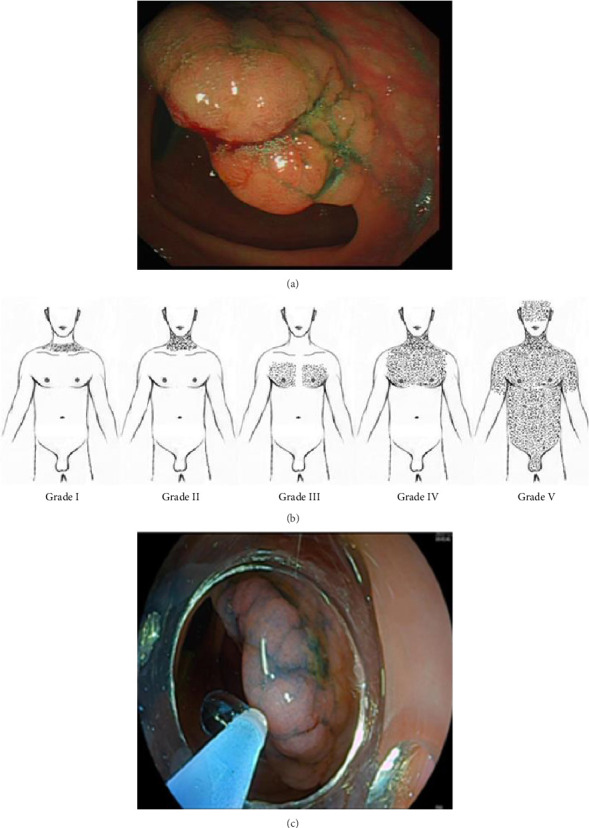
(a) Colonoscopy shows a cauliflower-like polyp growing in the annular cavity of the posterior wall of the ascending colon, 11 mm in size, with irregular in shape, and rough in surface. (b) Grade V subcutaneous emphysema per Aghajanzadeh classification [2]. The shaded area represents the extent of subcutaneous emphysema. (c) Electrocoagulation was performed to remove the polyp without any complications.

The patient of the case was a 55-year-old female who was ordinarily in good health. The patient was admitted to the hospital with abdominal pain for 2 months, and a colonoscopy revealed a cauliflower-like mass in the annular cavity of the posterior wall of the ascending colon with a rough surface, occupying 1/2 of the intestinal lumen (Figure 1(a)). Colonoscopy biopsy pathology showed low-grade tubular adenoma. The patient was informed of the pathologic findings of her lesion and of possible treatment options. The mucosa was otherwise normal. The lesion was removed by ESD via a second colonoscopy after obtaining informed consent of the patient.

**Figure 2:**
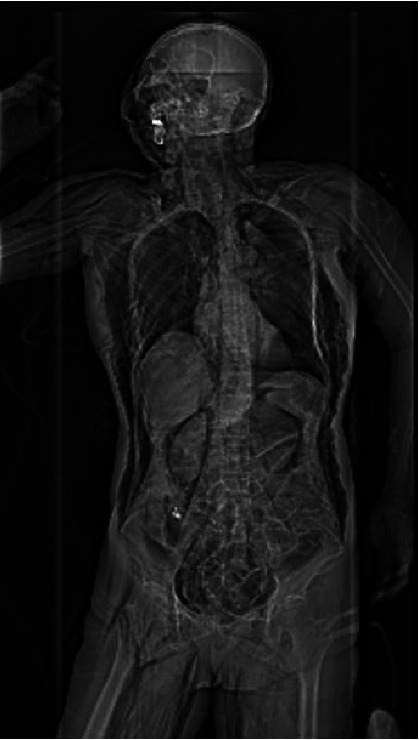
CT scanogram showing subcutaneous emphysema in the face, neck, chest, abdomen, and upper thighs.

**Figure 3:**
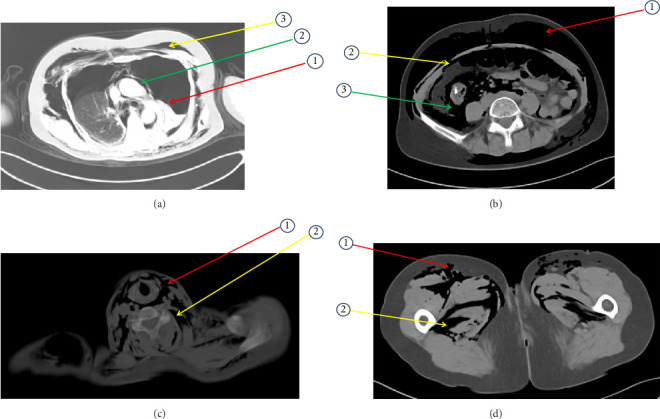
(a) Axial CT (lung window) showing: ① bilateral pneumothorax with near-total collapse of the left lung (red arrow); ② pneumomediastinum anterior to the ascending aorta (green arrow); ③ extensive subcutaneous emphysema in the left chest wall (yellow arrow). (b) Axial CT (abdominal window) demonstrating: ① extensive subcutaneous emphysema in the anterior abdominal wall (red arrow); ② pneumoperitoneum (yellow arrow); ③ pneumoretroperitoneum in the bilateral perirenal spaces (green arrow). (c) Axial CT of the neck showing: ① subcutaneous emphysema in the platysma and superficial fascia (red arrow); ② gas dissecting along the strap muscles and deep cervical fascia (yellow arrow). (d) Axial CT through the upper thighs showing: ① extensive subcutaneous emphysema (red arrow); ② gas extending along the fascial planes in the anterior and medial compartments of the thigh (yellow arrow).

The patient underwent colonoscopic tumor resection under monitored anesthesia care (MAC) with air insufflation for ESD of the mass. After endoscopic staining with methylene blue, the opening of the glandular ducts of the ascending colon was seen clearly, and the elevated mucosa was injected with a disposable syringe needle with a positive elevation sign, and then the lesion was completely peeled off layer by layer with electrocoagulation to extend to the muscularis propria. About 3 h after the surgery started, the patient suddenly developed emergent respiratory distress, manifesting as acute agitation and tachypnea (respiratory rate of 28 breaths/minute), coinciding with oxygen desaturation (SpO_2_ 88%–90%). It was noteworthy that the hemodynamic parameters remained within normal range (heart rate 78 beats/minute, noninvasive blood pressure 125/80 mmHg). Immediately initiated high-flow nasal cannula (HFNC) at FiO_2_ 1.0, flow rate 10 L/min. Achieved target SpO_2_ ≥ 92% within 8 min. The patient maintained spontaneous breathing throughout the procedure without endotracheal intubation. Meanwhile, physical examination by the anesthesiologist revealed extensive SCE involving the facial region extending caudally to encompass the thoracic, abdominal, and bilateral femoral regions, classified as grade V according to the Aghajanzadeh [2] (Figure 1(b)) grading system. Auscultating the lungs, the breath sounds in the left lung were absent, and the breath sounds in the right lung were weakened. Notably, the clinical evaluation demonstrated the absence of peritoneal irritation signs. The team decided to suspend CO_2_ insufflation and switch to low-flow manual gas injection. The endoscopist conducted an immediate endoscopic reassessment and did not observe any defects in the intestinal wall. The surgical timeline was strategically optimized through multidisciplinary team (MDT) involvement of thoracic surgeons, anesthesiologists, and endoscopists' clinical evaluation. Considering that the patient was hemodynamically stable (mean arterial pressure > 65 mmHg), the tumor lesion had been nearly completely dissected, and the termination of the surgery could lead to open perforation of the intestinal lumen, the MDT team decided to expedite the completion of the critical steps under continuous monitoring based on the risk-benefit analysis. Finally, the patient's colon tumor was successfully removed, with nine internal endoclips placed (Figure 1(c)). The entire intervention took 3 h and 20 min to be completed. Digital rectal examination showed no palpable sphincteric laxity or perirectal crepitus. Emergency arterial blood analysis results were as follows: pH 7.19, PO_2_ 70 mmHg, PCO_2_ 52.9 mmHg, SO_2_ 89.1%, actual base excess (ABE) -8.7 mmol/L, lactate 0.3 mmol/L. It suggested hypoxemia and respiratory acidosis with metabolic acidosis. After 100% oxygen therapy, a whole-body CT scan showed massive bilateral pneumothorax (approximately 95% of the left thoracic cavity and 80% of the right thoracic cavity), pneumomediastinum, pneumoperitoneum, and SCE (gas extended upward to the maxillofacial region, neck, and chest and downward to the low back, pelvis, lower rectum, and bilateral thigh roots) (Figures 2, 3(a), 3(b), 3(c), and 3(d)). Emergency department laboratory tests resulted in a normal leukocyte count and C-reactive protein (CRP). After evaluation by thoracic surgeons, the compression range of bilateral pneumothorax was less than 30% and there were no tension signs (no jugular vein distension/tracheal offset), so it was preferred to evacuate about 500 mL of air from the left chest cavity urgently. Then a bilateral chest drain was placed to continue to drain a moderate amount of gas, after which the patient's symptoms of dyspnoea and tachypnoea improved significantly, and her SPO_2_ rose to 98%. It took about 28 min from the emergency CT scan to the placement of the drainage tube. Re-arterial blood gas analysis 3 h later results showed: pH 7.38, PO_2_ 145 mmHg, PCO_2_ 31.0 mmHg, SO_2_ 98.2%, ABE -5.7 mmol/L, and lactate 1.7 mmol/L, suggesting metabolic acidosis with partial respiratory compensation. The respective laboratory test outcomes were CRP remained normal, leukocytes were 14.22 k/μL, and neutrophils were 93.3%, indicating the possibility of intra-abdominal infection.

Upon detection of extensive emphysema, the endoscopist did not perform active colonic decompression (e.g., rectal tube placement or endoscopic suction), and the emphysema was considered due to microperforation. Furthermore, there was no intra- or retroperitoneal fluid collection on CT. Therefore, following conservative management including oxygen therapy, strict fasting, intravenous hydration, analgesia, antibiotics, and an indwelling catheter in the anal canal, the patient's symptoms improved further. The patient was also encouraged to start respiratory exercises. On postoperative day 4, the patient's bloating had noticeably subsided, and stepwise enteral nutrition was initiated. Postoperative day five, CT fistulography excluded bronchopleural fistula. Over the next few days, chest and abdominal X-ray were repeated, and the amount of free air gradually decreased. On the 12th postoperative day, chest X-ray confirmed complete re-expansion of both lungs. Therefore, bilateral chest drainage tubes were removed without causing any problems. On the 14th day after surgery, the patient had a smooth recovery and was discharged from the hospital. The patient was reviewed 2 months after surgery and recovered well without complications.

## 3. Discussion

Colonoscopy is an effective means for the diagnosis and treatment of gastrointestinal mucosal lesions, which is technically difficult, time-consuming, and challenging for endoscopists. Bleeding and perforation are the most common adverse events in clinical practice [3, 4]. These cases are extremely rare. According to reports, the incidence of colonic perforation during colonoscopy varies, ranging from as low as 0.03% for diagnostic colonoscopy to as high as 2.14% for therapeutic interventions involving polypectomy [5, 6]. According to the consensus on the imaging assessment of pneumothorax [7], CT surpasses X-ray films in precisely delineating anatomical stratification (including the degree of lung compression and mediastinal structure displacement) for complex pneumothorax complicated by mediastinal displacement. Thus, in our case, postsymptom onset urgent CT scans revealed bilateral massive pneumothoraces, mediastinal emphysema, pneumoperitoneum, and extensive SCE throughout the body. While CT remains the gold standard for characterizing complex gas dissemination patterns, portable chest radiography (CXR) retains critical utility in resource-limited settings. In such cases, chest and abdominal X-rays may reveal pneumoperitoneum, pneumothorax, or SCE, prompting timely referral for further imaging or intervention.

An important observation from this case is that no bleeding or macroscopic perforation was noted during the procedure, suggesting microperforation as the likely aetiology. According to a Korean study, perforations during ESD are categorized into macroscopic and microscopic types. Macroscopic perforations can be readily identified through endoscopy. In contrast, microscopic perforations, which lack visible signs and are typically undetectable, occurred in only 2.1% of the 823 ESD procedures for gastric lesions in the article [8].

Although both types of perforation may result in extensive tissue damage and diffuse peritonitis, increasing age, female gender, barotrauma, thermal injury, depth of lesion infiltration into muscularis mucosa, and prolonged procedure (> 2 h) may all be risk factors for microperforation [9, 10]. In this clinical case, the female patient presented with a mass in the posterior wall of the ascending colon with a relatively weak intestinal wall structure and a long surgical procedure (3 h 20 min), factors that are particularly important in the evolution of surgical complications. We believe that one of the possible inducements of microperforation lies in the operation details of submucosal injection with a needle during surgery, which may inadvertently cause microinjuries in the weak intestinal wall area due to the extremely high level of delicate dissection required when accessing the submucosal layer. Another potential factor that should not be overlooked is that the continuous use of electrocoagulation during the procedure may cause mild thermal damage to the intrinsic muscular layer of the intestinal wall, which may result in transmural air leakage. Diagnosis of this microperforation complication depends on a CT scan to clearly detect the presence of free air in the abdominal cavity [11, 12].

Once colonic perforation occurs, intraluminal gas may escape into the peritoneal cavity and/or retroperitoneal space. In this case, the ascending colon lesion location suggests retroperitoneal microperforation. This is the primary route explaining the observed complications. Gas escaping retroperitoneally can readily track along fascial planes cephalad into the mediastinum (causing pneumomediastinum) and subsequently rupture into the pleural spaces (causing bilateral pneumothorax). Free intraperitoneal gas can also dissect along tissue planes, potentially contributing to widespread SCE extending to the neck, face, and thighs [6, 13, 14]. While congenital fascial defects or weak areas remain a theoretical pathway for gas migration [6, 14], the retroperitoneal origin provides the most plausible explanation for the extensive mediastinal and pleural involvement observed here, consistent with established anatomical continuities.

Symptoms of perforation were not detected until several hours later. Previous studies have revealed that 52% of perforations are detected immediately or within 1 h, 29% are found between 1 and 24 hours, while 19% are detected 24 hours or more after the procedure [15, 16]. In the case presented, it is difficult to say when exactly the microperforation occurred; it is only possible to say that no clinical signs were apparent clinically until 3 h into the procedure. This means that perforation of the ascending colon may occur during electrocoagulation polyp removal, coupled with progressive diffusion of gas caused by continuous intestinal aeration leading to surgical emphysema in the anatomical muscle planes.

Insufflation of air may be another cause of discomfort during and after colonoscopy in our case. Studies comparing the use of air versus carbon dioxide insufflation during colonoscopies have shown that the use of carbon dioxide significantly reduces abdominal pain and discomfort during and up to 24 h after the procedure, as carbon dioxide is rapidly absorbed through the intestines and eliminated by the lungs [17]. Therefore, CO_2_ should be routinely used instead of air for colonoscopy. However, specific research regarding whether the use of carbon dioxide insufflation can more timely detect perforations is lacking.

In our clinical case presented, the diagnosis was made through the clinical history, including the time of symptom occurrence and the clinical manifestations during the physical examination, which were presented as crepitus in the face, neck, chest, abdomen, back, and bilateral thigh areas. Subsequently, CT imaging identified SCE, pneumoperitoneum, bilateral pneumothorax, etc. The definitive management of gastrointestinal perforation requires precise localization and repair of the defect. Concurrent SCE may necessitate decompressive interventions—including subcutaneous needle aspiration, infraclavicular “blowhole” incisions, drainage catheter placement, or cervical mediastinotomy—to facilitate gas evacuation from body compartments. Upon identification of extensive SCE, it should be stratified according to the anatomical scope of emphysema as proposed by Aghajanzadeh [2] et al., including (1) base of the neck, (2) all of the neck area, (3) subpectoralis major area, (4) chest wall and all of the neck area, and (5) chest wall, neck, orbit, scalp, abdominal wall, upper limbs, and scrotum (Figure 1(b)).

Treatment of SCE remains controversial because therapeutic strategies primarily necessitate addressing the underlying etiology. Mild cases of SCE may only require strict bed rest, multimodal analgesia, and high-flow oxygen therapy. The latter modality accelerates nitrogen resorption through facilitated gas diffusion gradients according to Henry's law. Conservative approaches should be complemented by patient reassurance with vigilant clinical monitoring of disease progression. In severe presentations where SCE precipitates compromised respiratory mechanics (e.g., impending ventilatory failure), cutaneous necrosis, or orbital compartment syndrome threatening visual acuity, invasive interventions become imperative. Surgical decompression via subcutaneous catheterization or infraclavicular “blowhole” incisions may be instituted to mitigate tissue pressure gradients. Escalation to thoracic surgical consultation is warranted for cases complicated by concurrent pneumomediastinum or tension physiology [2].

In our patient, dyspnea and abdominal distension were the sole clinical manifestations. CT imaging failed to identify a definite perforation site and the absence of peritonitis or hemodynamic instability justified a conservative strategy. After multidisciplinary-team discussion, protocol-based interventions were implemented, including high-flow nasal cannula oxygen, bilateral chest drains, and structured incentive spirometry. Respiratory symptoms and extensive SCE resolved progressively, and serial imaging showed spontaneous absorption of pneumoperitoneum, obviating the need for surgery.

## 4. Conclusion

In summary, our case report describes a rare complication of gas extravasation during ESD of a colonic polyp, manifesting as bilateral pneumothorax, mediastinal emphysema, pneumoperitoneum, and generalized SCE, highlighting the possible pathogenesis of the anatomical relationship by which emphysema migrates to different topographic levels. The purpose of presenting this case is to alert the physician who performs these interventions to suspect this complication in a timely manner. If this rare complication occurs, the site of perforation should be actively sought and managed accordingly. In settings where CT is unavailable, serial plain radiographs may serve as an alternative for monitoring gas resolution. This case also underscores the rapid collaboration between anesthesiologists, radiologists, and thoracic surgeons, which led to accurate diagnosis and timely intervention. Based on advanced imaging techniques, effective collaboration between multidisciplinary physicians enabled the patient to receive thoracic catheterization and conservative supportive treatment, avoiding surgery and achieving good results.
